# An observational study of pediatric healthcare burden in Angelman syndrome: results from a real-world study

**DOI:** 10.1186/s13023-019-1210-6

**Published:** 2019-11-04

**Authors:** Nasreen Khan, Raquel Cabo, Wen-Hann Tan, Regina Tayag, Lynne M. Bird

**Affiliations:** 1 RWEC LLC, 73 Walsingham, Mendham, NJ 07945 USA; 2 Division of Genetics & Genomics, Boston Children’s Hospital; Harvard Medical School, 300 Longwood Avenue, Boston, MA, Boston, MA 02115 USA; 3 PROMETRIKA, LLC, 100 Cambridgepark Drive, 2nd Floor, Cambridge, MA 02140 USA; 4 Department of Pediatrics, San Diego; Clinical Genetics / Dysmorphology, Rady Children’s Hospital San Diego, University of California, 3020 Children’s Way #5031, San Diego, CA 92123 USA

**Keywords:** Healthcare economics, Healthcare resource utilization, Rare disease, Disease burden

## Abstract

**Background:**

The objective of this study is to describe variations in the healthcare resource utilization (HRU) among individuals with Angelman syndrome (AS) over the first 12 years of life. Data for this study were drawn from the AS Natural History study (ASNHS), which is an observational study on the developmental progress, behavior, and medical morbidity of individuals with AS conducted over eight years. Caregiver-reported information on hospitalization, surgery, and medication utilization was used to assess HRU. Repeated measures mixed effect models were used to assess the relationship between age and probability of hospitalization, surgery, and prescription medication utilization.

**Results:**

Mean age at study enrollment was 6 years of age and both sexes were equally represented. The mean number of visits per participant was three. Results from this study suggest that individuals with AS have a high HRU burden. Hospitalization and surgery burden were highest in the first year of life. Use of medications for seizures and sleep disturbance increased over time.

**Conclusions:**

The study highlights the significant healthcare burden among individuals with AS. Future studies that estimate cost and caregiver burden associated with AS are needed to assess the lifelong economic impact of AS on families and healthcare system.

## Background

Angelman syndrome (AS) is a rare, neurodevelopmental condition characterized by severe impairment in behavior, motor function, sleep, and cognition. There are no approved treatments for AS and the current goal is management of comorbidities and symptoms [1]. AS has an impact on overall health, severely limiting activities of daily living and individuals require lifelong support from a network of specialists and caregivers.

There is limited information on the healthcare burden of individuals with AS and how it varies with age. In a study of 34 individuals with a mean age of 21.6 years, [2] Thomson et al. found that individuals with AS had a high hospitalization burden (median of 5.5 hospitalizations per person) and the most common reasons for hospitalization were seizures, gastrointestinal disorders, and dental work. Another study found that the most common reasons for hospitalization were dental care, seizures, orthopedic problems, and acute respiratory disorders [3]. Our previous analysis using data from baseline visits of the AS Natural History Study (ASNHS) found that more than 60% of individuals had a history of at least one hospitalization from birth to enrollment into the study [4]. The most common reasons for hospitalizations were seizures, lower respiratory infections, and surgery. The most commonly used medications were those for treatment of seizures, gastroesophageal reflux disease, sleep, and behavioral disorders. In addition, our study showed that individuals with AS had high utilization of supportive therapies, such as early childhood intervention and physical, occupational, and speech therapies to promote development.

While the above studies have established the significant healthcare burden for individuals with AS, there are no published data on how this burden changes with age. The primary aim of this study is to describe how healthcare resource utilization (HRU) varies with age in the pediatric population with AS in the United States (US). By documenting the HRU among children with AS, we will begin to characterize the unmet needs for this population, which may help determine how resources should be allocated for the management of this chronic condition.

## Methods

### Data

The ASNHS gathered longitudinal data on the developmental progress, behavior, and medical morbidity of individuals with AS from 2006 to 2014 [5]. The study was conducted by the Angelman, Rett, and Prader-Willi Syndromes Consortium of the NIH Rare Diseases Clinical Research Network (ClinicalTrials.gov Identifier: NCT00296764). Individuals with AS were recruited at six study sites across the United States. Inclusion criteria included a molecular or clinical diagnosis of AS, and age between 1 day and 60 years. A total of 311 individuals were enrolled in the ASNHS. For the purpose of this analysis, only 302 individuals with molecularly confirmed AS and no concomitant medical condition (similar intellectual disability) were included.

At the baseline visit and at each annual follow-up visit, data were collected through interviews with the caregiver who was present at the visit about the participant’s previous and current medical history and developmental progress since birth. With respect to HRU, the dates of, and indications for, any hospitalization or surgery, and length of stay (LOS) were recorded. Information was also collected on the use of prescription and non-prescription medications, including the reasons for using each medication and the duration of use.

The date of event (e.g., hospitalization) and date of birth were used to calculate the age at time of event, if age was not directly reported. If the event date was completely missing, the date of visit at which the event was reported was used to calculate the age at time of event, provided that no more than one annually scheduled visit was missing immediately prior to the visit reporting the event. If there was more than one missing annual visit immediately prior to the visit reporting the event, then age at time of event was considered missing and not included in the analysis. As we could not determine whether a given surgery was performed in an outpatient or inpatient setting, hospitalization and surgery data were not considered mutually exclusive. In addition, length of stay (LOS) was defined as the number of nights in a hospital, with a minimum of one overnight stay in the hospital. Finally, analyses were restricted to individuals 12 years of age or younger due to small sample sizes for older age groups.

### Statistical analysis

Descriptive statistics of the resource utilization in the sample are presented by age. For continuous variables, the mean and standard deviation (SD) are presented. For categorical variables, frequencies and related percentages are presented. To analyze how healthcare utilization changes with age, a mixed model for repeated binary measurements was used. Separate models were fitted to estimate the probability of hospitalization, surgery, and prescription medication utilization. Models included fixed effects for intercept, age, molecular genotype, and genotype-by-age interaction, and random effects for intercept and slope. Molecular genotype was defined as a binary measure where individuals with a deletion genotype were categorized together and individuals with non-deletion etiology were categorized otherwise, as the reference category. Molecular etiology was added as a covariate since previous studies suggested that individuals with deletion genotype generally have a more severe course [5–9]. All statistical analyses were performed using SAS® Version 9.4 or higher for Windows.

## Results

**Fig. 1:**
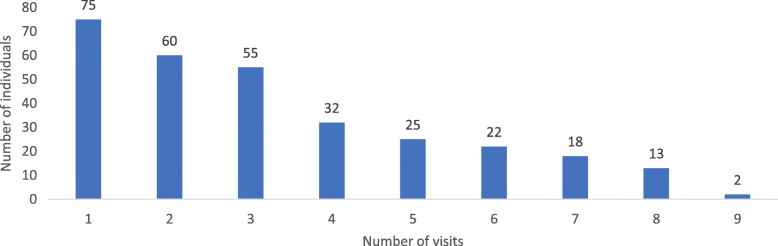
Number of individuals by number of visits, Notes: Number of participants with the stated total number of visits; participants who only had the baseline visit had “1 visit”. Follow-up visits occurred approximately annually for participants

Table 1 shows basic demographic information and available data at the time of enrollment. Mean age at AS diagnosis was two years and 48% were male. Most of the participants (62%) were less than five years old at the time of enrollment. On average, individuals had approximately three annual visits, including the baseline visit during which historical data were collected. Figure 1 shows the number of participants with the stated total number of visits. Approximately, 75% of individuals had at least two visits, 55% had at least three or more visits, and 37% had four or more visits.

**Table 1:** Baseline descriptive data and data availability in this study

*Variable*	*N = 302*
Age at diagnosis, years (mean, SD)	2 (3)
Male (n, %)	145 (48%)
Age at baseline, years (mean)	5.5 (5.9)
0–1 year (n, %)	53 (18%)
2 years (n, %)	61 (20%)
3 years (n, %)	37 (12%)
4 years (n, %)	35 (12%)
5 years (n, %)	20 (7%)
6 years (n, %)	11 (4%)
7 years (n, %)	8 (3%)
8 years (n, %)	11 (4%)
9 years (n, %)	14 (5%)
10 years (n, %)	9 (3%)
11 years (n, %)	7 (2%)
12 years (n, %)	9 (3%)
> 13 years (n, %)	25 (8%)
Number of visits, (mean, min-max)	3.3 (1–9)

Table 2 presents descriptive statistics on HRU by age. The occurrence of hospitalization was highest at 43% at age one year or younger, decreased over time, and was only 11% at age 12 years. There was a slight decrease (27%) in the mean number of hospitalizations over time from 1.5 hospitalizations by age one to 1.1 hospitalizations at age 12 years. Among those hospitalized, mean LOS was 6.5 days at age one year (SD: 8.9), 3.6 days (SD: 5.8) at six years and 1.5 days (SD: 0.71) at 12 years. Seizures and lower respiratory infection were the most common reason for hospitalization. Similarly, surgeries were more common in the younger patients: 29%, 9%, and 5% at age one year, six years, and 12 years, respectively. Tympanostomy tube insertion, strabismus correction, and tonsillectomy and adenoidectomy were the most frequent surgeries in this sample and were more common in infancy. Majority of individuals used one or more prescription medications and utilization increased with age (51% by age one year to 83% at age 9 years), after which a slight decline was observed. The mean number of prescription medication utilization increased by 26% between age one year and age 12 years, with the increase being non-linear. Use of anticonvulsant medications increased from 32% at age one year to 73% at age seven years and stabilized thereafter. Use of medications to treat GERD decreased from 27% at age one year to 13% at age six years and stabilized thereafter. Notably, there was an increase in the number of individuals using medications for behavioral and mental health indications, which increased from 1% in the younger years to 23% at age 12 years. Similarly, there was an increase in the number of individuals using non-prescription sleep medications: 18% by the first year of life, 37% at age five years, and 23% at age 12 years.

**Table 2:** Healthcare utilization among individuals with AS by age

Descriptive	0–1	2	3	4	5	6	7	8	9	10	11	12
N^1^	301	282	260	234	208	179	155	134	114	92	77	65
Hospitalization summary measures												
Had hospitalization, %	43	24	21	18	14	13	13	10	12	7	8	11
Hospitalization per year, mean	1.5	1.3	1.3	1.6	1.4	1.1	1.1	1.2	1.4	1.2	1.3	1.1
LOS, mean	6.5	3.1	2.5	2.6	4.7	3.6	3.1	2.7	2.9	1	3.4	1.5
Most common reasons for hospitalization												
Seizures, %	10	11	10	6	7	5	5	4	3	0	3	1
Lower respiratory infection, %	10	5	1	3	1	1	1	2	3	1	0	0
Surgery, %	5	3	2	2	1	0	1	2	0	0	0	0
Surgery summary measure												
Had surgery, %	29	21	15	17	9	9	14	8	9	4	8	5
Most common reasons for surgery												
Tympanostomy tubes, %	10	5	5	3	4	1	2	0	2	0	1	0
Strabismus, %	7	9	2	3	1	2	2	1	1	0	0	0
Tonsillectomy & adenoidectomy, %	2	2	5	5	2	2	1	0	1	0	0	0
Medication summary measures												
Number of prescription medications, mean	2.1	2.0	2.1	2.3	2.3	2.1	2.2	2.2	2.3	2.5	2.5	2.7
At least one prescription medication, %	51	59	69	71	76	78	81	81	83	77	78	77
Anti-epileptic drugs, %	32	50	61	64	67	69	73	71	72	71	70	69
Anti-gastroesophageal reflux medication, %	27	15	12	13	12	13	11	12	13	10	12	9
Asthma and Allergy medication, %	5	5	7	9	7	6	7	7	8	5	4	6
Antibiotic, %	3	1	4	2	1	1	2	1	1	2	0	2
Sleep medication, %	3	6	9	12	15	18	17	19	18	16	20	26
Psychotropic medication, %	1	1	2	6	9	9	13	15	17	22	20	23
Other, %	2	1	6	5	6	5	8	6	5	7	10	11
Number of non-prescription medications, mean	1.6	1.9	1.8	1.9	1.9	2.0	2.0	2.1	2.1	2.2	2.1	1.8
Asthma and Allergy, %	3	5	5	6	7	7	8	5	5	5	7	8
Laxative, %	11	14	15	15	14	14	18	19	17	19	16	19
Sleep medication, %	18	30	31	35	37	33	33	34	31	29	25	23

Notes: ^1^Age is rounded down to the nearest year. The event counts for each age are based on the participants reported age at a specific event. Participant are included in the overall N (denominator) for each age group until their age at last visit (the oldest age recorded). Participants are only counted once per age. For ages 0–1 Year, the sum of the number of unique prescription/non-prescription medications taken at age 0 and at age 1 is summarized

Table 3 presents estimates from repeated measure mixed effect models for the probability of hospitalization, surgery, and medication use. Results from these models are consistent with the descriptive information presented in Table 2. Estimates of the effect of age indicate that the odds of hospitalization or surgery decreased with increasing age. In contrast, based on the model, an increase in age was associated with an increase in the odds of prescription drug utilization in our sample. Specifically, the model suggests that the probability of hospitalization at age one year among those without a deletion was 0.20, and 0.05 at age 12 years. In contrast, the probability of use of prescription medication among those without deletion was 0.14 at age one year and 0.99 at age 12 years, and among those with a deletion, it was 0.54 and 0.99 at age one year and 12 years respectively.

**Table 3:** Repeated measures mixed effect model of probability of use of healthcare utilization

	Hospitalization	Surgery	Prescription medication
N	298	297	297
Number of observations	2384	2387	2387
Intercept	−1.222* [0.1740]	− 1.599* [0.1936]	−2.628* [0.4812]
Age	−0.149* [0.0452]	−0.079 [0.0436]	0.822* [0.1697]
Molecular genotype	0.384* [0.1958]	0.176 [0.2143]	1.166* [0.5341]
Age*Molecular genotype.	−0.056 [0.0497]	−0.078 [0.0472]	0.808* [0.2230]

Notes: A repeated measures mixed effect binary model was assessed for each healthcare resource. Each column represents a separate model and the column heading indicates the dependent variable. Each model predicts the probability of an event (e.g., hospitalization, surgery or use of prescription medications) controlling for molecular genotype. N refers to unique number of individuals and the number of observations refers to the total number of years of available data for all individuals for an analysis. Standard errors are in parenthesis. The main coefficient of interest is age and it depicts how utilization changes with age. Based on the models above, a positive coefficient for age suggests that utilization increases with an increase in age and a negative coefficient suggests a decrease in utilization with an increase in age

*Significant at *p* < 0.05

## Discussion

AS is a rare condition with an estimated prevalence of 1 in 12,000 to 1 in 20,000 in the US [10]. The most consistent features are global developmental delay marked by intellectual disability, seizures, severe speech impairment, behavior problems, and sleep disturbance, but the presentation and severity of symptoms varies among individuals and changes with age [11–13]. To the best of our knowledge, this is the first study to present the healthcare burden associated with AS, from infancy to age 12 years.

The ASNHS is a large-scale longitudinal study of individuals with AS in the US designed to improve knowledge of the condition and investigate associated morbidity across ages. Individuals in the analyzable sample were younger than 12 years with a mean age of six years and equal sex distribution. On average, we had three years of data for each individual. Our analyses suggest an overall high HRU in this population especially among younger children between 0 and 1 years of age. Hospitalization, surgeries and use of prescription medications to manage various symptoms were common. Our data suggests that younger children, often in the first year of life, tended to experience more surgeries, hospitalizations, and longer hospital stays than older children. The use of prescription medications increased with age in this cohort and by age six almost 80% of children were using at least one medication.

Our analyses support the heterogeneity of the condition. It appears that the various symptoms were managed through a combination of hospital-based interventions and prescription medications. Seizures are one of the most common symptoms associated with AS [11], and antiepileptic drugs (AEDs) are the accepted first-line treatment for managing seizures in these individuals. In this study, we found that AEDs were the most commonly used class of medication, regardless of age, consistent with the high incidence of seizures in this population.

Medications to treat sleep disturbance were the other common category. Sleep difficulties may manifest as increased sleep latency, decreased total sleep time, abnormal sleep-wake cycle, and frequent nocturnal awakening [11]. Some studies suggest that sleep disturbances are more common among young children 2 to 9 years of age and improve with age, while others report that they continue into adolescence and adulthood [1]. We found that use of sleep medications increased with age. Use of melatonin, a commonly used non-prescription medication, was 18% in the first year, peaked at age 8 (34%) and continued to be high into early adolescence (23 to 34%).

Individuals with AS may have behavioral issues, such as hyperactivity, impulsivity, and aggressive behaviors [11]. We observed increasing use of behavioral/psychiatric (psychotropic) medications with age. Use of medication to treat GERD was highest in the first year of life, after which use decreased, mirroring the typical age when this issue is most problematic.

This study has several limitations. The population was skewed towards individuals younger than five years old and the mean number of data points per individual was only three. The ASNHS study did not record any information on use of outpatient care (e.g., visits to primary care physicians, neurologists, geneticists, psychiatrists, and other specialists). While not as costly as hospitalization, outpatient care tends to be one of the largest volume drivers of healthcare burden. We were unable to determine whether the reported surgeries were performed in an outpatient or an inpatient setting. Since the data used in these analyses were collected via caregiver (either primary or informal) interviews, the HRU was likely underreported due to recall failure and should be considered a minimum estimate, particularly true for medication use. In addition, we are using the event dates to create longitudinal history of healthcare utilization and misreporting is likely to be higher for medication use and as such the medication results should be interpreted in the light of these limitations. Replication of the findings in this study using claims data or administrative sources will help to corroborate these results.

## Conclusions

In summary, this is the first study to describe how the HRU varies with age in children with AS. We have shown overall high HRU use as well as different types of HRU, to manage a constellation of heterogeneous symptoms and co-morbidities. The high healthcare burden for these individuals and their families can be demonstrated by the high hospitalization and surgery rates, especially in the first year of life. We also described how the use of medications increased with age, especially for conditions such as seizures, sleep disturbance, and behavioral issues. Future studies are required to provide a holistic view on the HRU use in both children and adults and evaluate the overall impact on caregivers and the healthcare system.

## Data Availability

Derived data that can help generate results can be provided from the corresponding author on reasonable request.
